# Medical device regulation (MDR) from a dental perspective

**DOI:** 10.3389/fdmed.2023.1155820

**Published:** 2023-03-23

**Authors:** Dirk Mohn, Matthias Zehnder

**Affiliations:** ^1^ Linio Biotech Ltd., Helsinki, Finland; ^2^ Conservative and Preventive Dentistry, University of Zurich, Zurich, Switzerland

**Keywords:** EUDAMED, notified body, ISO, medical technology, MDR, MDD

## Abstract

A new regulation for the marketing and application of medical devices has become applicable in the European Union as of May 2021. This regulation is termed EU 2017/745 or Medical Device Regulation (MDR). Initially published and entered into force in 2017, it replaces the former Medical Device Directive 93/42/EEC (MDD), but is still under amendment. The implication of this legislation have broad effects on manufacturers, importers, distributors, users of medical devices, and patients. This article discusses the MDR from the dental perspective. As is illuminated in this text, the MDR will create more red tape for industrial players to get their products CE (Conformitée Européenne) marked, and more documentation work for dentists. This also means that smaller companies acting out of Europe are affected in a disproportionally negative manner compared to their globally acting counterparts. The MDR could and most probably will result in a considerable reduction and price increase of the products that are available to European dentists. Moreover, the MDR could create a rift between dental materials scientists working at universities and the dental industry, because the latter now has to direct more money towards regulatory affairs rather than product development or innovation. On the other hand, the MDR may also act as an antetype for similar regulations in other parts of the world, and could offer new career opportunities for individuals in dental materials research, especially in the regulatory field.

## Introduction

A new regulation for the marketing and application of medical devices has become applicable in the European Union as of May 2021. This regulation is termed EU 2017/745 or Medical Device Regulation (MDR) and opens a new chapter in the medical device rulebook. When entering into force, an EU regulation by definition automatically and uniformly applies to all EU countries, without a requirement to be transposed to national law. Non-EU countries in search of EU agreements such as Switzerland and Great Britain adopted their medical device legislation as well. Switzerland has named its legislative text Medical Devices Ordinance and is referring in a vast majority of the articles to the MDR. Similarly, after Great Britain left the European Union, an adopted United Kingdom MDR was implemented with almost no deviations from the EU MDR (1).

The original intention of the MDR was to “establish a robust, transparent, predictable and sustainable regulatory framework for medical devices that ensures a high level of safety and health while supporting innovation”. The fundamental changes in the MDR from the formerly imposed directive, the Medical Device Directive (MDD, 93/42/EEC), were triggered by the fraudulent use of technical grade silicone for breast implants, the PIP (poly implant prothèse) scandal (2) and adverse events related to metal hip implants (3). Both episodes forced the legislators to implement stricter rules for medical device certification. Better surveillance after market approval and stricter quality controls with more clinical evidence before medical device certification were requested (4). Moreover, the MDR covers a broader spectrum of applications than the MDD, as it explicitly includes all products for the cleaning, sterilization, or disinfection of other medical devices (Table 1). In its essence, the MDR requests an extensive product data set including clinical data to document the whole life cycle of a medical device. Product tracing is made possible *via* the implementation of a unique device identifier (UDI, see chapter on EUDAMED database below).

**Table 1:** I New MDR features in their attempt to cover all aspects in a product's life cycle and to include clinical data.^a^

• More thorough pre- and post-market assessments by manufacturers • Expansion of technical documentation and labeling • Notified bodies need to fulfill more criteria and show clinical competence • Coverage of devices for non-medical purpose and cleaning/sterilization of other devices • Reclassification of many medical devices and introduction of new classes, e.g. class Ir, reusable surgical devices • Consulting by independent body for some high-risk (Class III) devices • Product tracking: unique device identifier (UDI) and EUDAMED database

^a^ All MDD requirements remain intact.

Despite all its potential benefits for patients, the new regulation has already been heavily criticized by medical device stakeholders for being unfair to small and medium-sized companies, and thus effectively killing medical device innovation in Europe (5, 6). By May 2027, the European Commission therefore wants to perform a comprehensive evaluation of the implemented MDR (7).

The MDR application date was in May 2021, after which every device needed to comply with this regulation. A transition period, during which MDD devices (legacy devices) can still be marketed, will end in May 2024. However, certain MDR requirements have to be followed by the manufacturers and other economic operators already (Art. 120, MDR) (8). At the time of finishing this article (January 2023), the European Commission proposed an extension of the transition period “based on the input from national experts and stakeholders” (9). At the core of this problem is the fact that there are too few accredited third-party certification bodies, the so-called notified bodies, which are legally capable to assess the MDR conformity of medical devices before being made available on the market. This has already resulted in product shortages and increased financial pressure on health-care institutions (10). To counter-act these problems, the potential European Commission extension aims to prolong the transition period for devices not presenting any unacceptable risk to health and safety and not having undergone any significant changes in design from the MDD (9). With this proposal the EU has admitted that there is a risk of device shortage. It has also become apparent that the rate of MDD certificate expiration is faster than the issuance of MDR certificates. Hitherto, most of the issued MDR certificates have merely been issued for low-risk devices. However, whether this proposal regarding an extended transition period will gain political acceptance is not yet clear. The EU Parliament is supposed to vote on the extension in the beginning of February 2023, and the EU Council will follow suit. It could take until Spring 2023 to transpose this extension into legislative text and to be approved by the EU Parliament and EU Council. Nevertheless, the threat of a device shortage that would affect patient care is real and cannot be taken lightly.

From a dental perspective, a few weeks before the proposal of the EU Commission, the Council of European Dentists (CED) released a statement also expressing their deep concern regarding the current lack of notified bodies responsible for device certification. This according to the CED puts the marketing of re-certified devices in jeopardy (11).

In summary, the MDR has created some turmoil, and apparently has not yet reached the minds of everybody that could be affected by it. This article discusses direct and indirect MDR implications for dentists and dental researchers, i.e., the core readership of this journal. As the new regulation primarily targets the medical device industry, the main changes imposed on manufacturers are highlighted, and the resulting real and potential implications on dentists and dental researchers are discussed.

## Background

The authors of this article are a former scholar in dental materials who now works for a medical device start-up company (DM) and a senior university researcher focusing on the development of dental materials and diagnostic tools (MZ). Together they founded their own company, smartodont llc, to promote technology transfer from university into the dental industry. This text reflects their joint approach to the topic, hands-on experience with the regulatory approval of products, and information gained in specific courses. The authors are aware that the MDR consists of many more details and referrals to other documents that cannot be covered in this text. It was their attempt to discuss the, in their view, most relevant parts for dentists and dental researchers rather than the whole process for MDR compliance.

## Notified body requirements

To sell their merchandise in the European Union (EU), manufacturers of medical devices need to get a CE (Conformitée Européenne) mark on their products. This mark indicates that a product complies with EU regulations and can be commercialized across the EU Member States, provided that it also complies with national registration and language requirements. To obtain this mark, a third-party certification body, the notified body, has to be employed by the manufacturer. The notified body acts as an intermediary between the authority and the manufacturer to ensure conformity of medical devices with the legal framework. Under the MDR, among other new tasks, notified bodies have to assess the Clinical Evaluation Report of each device under scrutiny and, thus, are in need of qualified experts (see also below under RESEARCH IMPLICATIONS). Currently (January 2023), there are merely 37 MDR-accredited notified bodies fully approved, as opposed to 75 such bodies in 2013 that could do conformity assessments under MDD.

The increased red tape and scarcity of available notified bodies have increased costs for medical device registration rather steeply for the manufacturers. In dentistry as in other medical fields, this is likely to lead to less products on the market and also increased prices for dentists when purchasing CE marked devices.

## Challenges for manufacturers

### Changes in classification

Within dentistry, medical devices can encompass a plethora of materials and applications (12). Class I devices for example, are impression materials, curing lights or examination gloves. Dental fillings, an implantable device as defined within the regulation, is Class IIa, whereas dental implants and their abutments are Class IIb. Products such as resorbable bone cements, endodontic filling materials including a drug, and animal derived bone graft substitutes all fall into the highest risk category (Class III). While developing and drafting the MDR, the EU proposed to implement rule 19 of the device classification (Annex VIII, MDR) to all devices that contain nanomaterial into the highest risk class (Class III). As various dental materials, such as fillings, pastes or cements contain nanomaterials in different amounts and sizes, this would have resulted in a high burden for dental manufacturers, of which a lot are small and mid-sized entities. It was strongly debated what should be classified as a “nanomaterial” and what the implications were. Importantly, nanoparticles in an aerosol have a completely different risk profile from bound and aggregated counterparts in for example, dental fillings (13). The Federation of the European Dental Industry (FiDE) published statements during the process of the nanomaterial definition and a justification why the used nanomaterials are of negligible internal exposure risk for the patient (14). Therefore, the MDR classification of a device containing nanomaterial now depends not only on the mere content of nanoparticles, but rather on the possibility for their release (15). The Medical Device Coordination Group (MDCG) of the European Commission provides a guideline including examples, in which dental fillings are classified as Class IIa devices (15).

It can thus be stated that the dental industry could prevent some original MDR plans for stricter classification. If imposed, such rules would have had a detrimental effect on the European dental materials industry. Nevertheless, what is new in the MDR as opposed to the MDD is that Class I devices have sub-Classes such as Class Ir, to which reusable surgical instruments such as dental curettes, mirrors belong. Although Class I, the involvement of a notified body for “the aspects relating to the reuse of the device, in particular cleaning, disinfection, sterilization, maintenance and functional testing and the related instructions for use” (Art.52, MDR) is required. Furthermore, the up-classification of software with a medical purpose as a medical device under MDR Classification Rule 11 is another controversially discussed topic (16). In general, software is now subsumed in higher classes as compared to the MDD. Software that influences treatment decisions is now classified as IIa, IIb, or even III if the treatment decisions could impact a patient's survival. Most diagnostic dental software falls into the Class IIa, as this is the default classification according to Rule 11.

### No grandfathering

One of the major MDR implications for manufacturers is the *no grandfathering* rule. All medical devices, which were approved under the MDD, have to be re-approved under the MDR. This means *new conformity assessments* for all medical devices currently circulating within the European Union Member States are required, complying with the new regulation and new rules. As of 25 May 2021, any device needs to comply with the MDR. A transition period, during which MDD devices can still be marketed, will end in May 2024 (current legal text). Already during this period (Art. 120, MDR), certain MDR requirements have to be followed by the manufacturers and other economic operators (8).

In general, to be able to market a medical device under the MDD or MDR, conformity has to be claimed. Depending on the risk class of a device the conformity can be claimed directly by the manufacturer (low risk Class I) or has to be assessed by an accredited notified body (mid and high-risk Classes IIa, IIb and III).

As indicated in the Introduction section of this text, one of the major threats of the approaching deadline in 2024 is the possibility that legacy devices (previously marketed under the MDD) could disappear because manufacturers will not be able to obtain their new MDR certification. Hence, a scarcity of devices could lead to a shortage in patient care in Europe. Whether this grim outlook in the medical field holds also true for dental medicine remains to be seen, as dentistry seems to have less high-risk devices compared to, for example, cardiology or orthopedics. At the time of writing this article, the EU Commission has proposed an extension until December 2027/2028 (depending on the risk class of the device) of the transition period (see Introduction), yet this proposal needs to be approved by the EU Parliament (9).

### EUDAMED database

The European database on medical devices (EUDAMED) is a new key element of the MDR to collect and process information about devices on the market. This openly available information comprises data on certification, clinical investigation, notified bodies, vigilance and market surveillance among others (17). EUDAMED should improve transparency, provide information for patients and healthcare professionals and facilitate the information flow between European Member States. Once the EUDAMED is fully functional (not at the time of finalizing this article, January 2023), the public should be able to view all relevant information related to a medical device. Economic operators, such as manufacturers, importers and European authorized representatives will be identifiable *via* a single registration number on a union-wide basis. Furthermore, all devices will have to feature a unique device identifier (UDI, Art. 27 MDR) to enable unambiguous tracking of devices on the market. Especially for Class III implantable devices, such as animal derived bone graft substitutes, health institutions shall store the UDI to enable tracking. Even for devices in lower risk classes, health institutions may be required by the EU Member State to store the UDI of a device with which they have been supplied.

EUDAMED will provide an easier access to device data for the dentists and the patient and could lead to a more educated treatment planning or outcome, while at the same time it could lead to more documentation work for the dentist in the form of storing information on devices which were used during a treatment.

### Implant card & SSCP

The implant card and additional information for the patient (Art. 18, MDR) is another new element, which has to be supplied by the manufacturer of an implantable device, that is, a device intended to be totally introduced into the human body and to remain in place after the procedure [see Art. 2 (5), MDR for more details]. Dental fillings, implants, or abutments therefore would all fall into this category. However, to impose this regulation on dental (restorative or endodontic) fillings would unnecessarily increase the burden on the manufacturers and dental healthcare professionals. Tooth crowns or dental fillings have therefore been exempted from the implant card obligation [Art. 18 (3), MDR]. Dental implants, their abutments, bone and soft tissue substitutes, on the other hand, do fall under the new implant card regulation.

In addition to the implant card, a summary of safety and clinical performance (SSCP) shall be provided by manufacturers of implantable and Class III devices (Art. 32, MDR). This summary will also be available to the public and shall be written in a way that it is easily understood by the intended user and, if applicable, by the patient. The SSCP has to feature a device description, references to previous generations, possible therapeutic alternatives, and a summary of the clinical evaluation [Art. 32 (2), MDR]. As only investigational or custom-made devices are exempted, devices which are readily available from the manufacturer are automatically included, as is for example a compliant and CE-marked root canal filling material. This could provide the dentist with further information without extensive literature search and could thus potentially influence his or her choice of material and treatment for a more beneficial outcome and patient safety.

### Clinical evaluation

To be able for medical device manufacturers to market their devices and to claim compliance with the MDR, the device in question has to meet the so-called *essential requirements*, which are general safety and performance requirements (GSPR) as laid out in Annex I (MDR General Safety and Performance Requirements). Part of meeting the GSPR is the evaluation of clinical data. This clinical data can originate from the device under evaluation or from an “equivalent” device. Under the MDD, claiming equivalence was a popular and somewhat cheap route to CE marking of copycat products related to devices that were not or not anymore covered by intellectual property, as no separate or new clinical investigation had to be undertaken. However, under the MDR *equivalence* has to be demonstrated for technical, biological and clinical factors. It has to be shown by the manufacturer that the evaluated device is not significantly clinically different to an equivalent device. Additionally, manufacturers have to have sufficient level of access to the technical data of a device to which they claim equivalence (18). Notified bodies interpret this as having full access to the technical file of the equivalent device. It goes without saying that, if that device originates from a competitor, this might rule out any equivalence claims for the device in question. This is one of the reasons why the new clinical evaluation causes a lot of extra work and costs to the manufacturers, especially those of high-risk devices.

The clinical data that is evaluated has to be of sufficient quantity and quality. Looking at the indications of a device, there might be lacks in terms of qualitative data for all the claims that a manufacturer might state in the intended use of a specific device. Furthermore, devices that have a large variety in size (e.g., stents of different lengths and diameters) could lack the necessary quantity in clinical data if certain extremely small or large variations are rarely used. This could lead to the reduction of claims due to lack of clinical data or the disappearance of such devices. In addition, old devices with a long history of clinical use but without sufficient clinical data might disappear as well.

Another part of the clinical evaluation is the post-market activities, i.e., events after a device was placed on the market. Within these activities come the requirements for post-market surveillance (PMS) and post-market clinical follow-up (PMCF). Both have to be performed by the manufacturers and have been installed to show the safety and performance in real life and over the whole lifecycle of a device. Again, new and high-risk devices have to be monitored especially closely.

In clinical dentistry case studies and case series, in which a novel treatment is done in conjunction with the application of an existing medical device, are reported frequently (19). In these studies, a medical device might not be applied according to its intended use and, thus, an off-label usage could be identified by a manufacturer during their PMCF activity. If systematic off-label use is identified, this could serve as clinical data (see also below under RESEARCH IMPLICATIONS). However, as recently stated by the Team-NB (the European association of medical devices notified bodies) this off-label application, while fulfilling the quantity for clinical data, could still lack the required quality, if no systematic approach has been undertaken. If a manufacturer would like to include the identified usage into the device's scope, a clinical investigation, according to MDR requirements, has to be performed (20).

### Private label manufacturing

**Figure 1:**
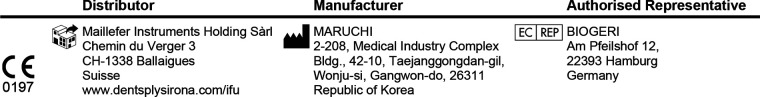
A screenshot of the Instructions for use of AH Plus Bioceramic showing the manufacturer as Maruchi and not Dentsply Sirona. It seems that this device, although following some MDR rules has not yet gone through MDR conformity assessment, as the current declaration of conformity is referring to the MDD.

Up to now and often unbeknownst to the dentist, there has been a concept called private or white label manufacturing. This implied that a manufacturer could produce for example, a root canal sealant, which was then marketed by three different companies, who used their own branding, labeling, marketing and pricing. Yet the actual content of the product was the same in all cases. Indeed, there are companies such as S&C Polymer that specialized in white label production in dentistry. Up to now (under the MDD), the original equipment manufacturer (OEM) went through the conformity assessment procedure with their own notified body and obtained a CE certificate. Subsequently, the OEM could offer their CE certified products to a private label manufacturer, who put their branding on the product, applied for CE conformity using the OEM certificate with their own notified body, without possessing the complete technical file of the product. The only condition for the private label manufacturer was to not modify the product from the OEM. This setup will not be possible anymore under the MDR, as the manufacturer has to have full access to the complete technical file. If a private label manufacturer still wants to market and sell a product, produced by an OEM, which has not been modified, the actual manufacturer (the OEM), has to be identified on the label and has to be required to be compliant with the MDR. If the OEM does not want to be responsible as the manufacturer, then the private label manufacturer has to be granted full access to the complete technical file and be responsible as the manufacturer according to MDR. Figure 1 shows an example of an endodontic sealer (AH Plus Bioceramic), marketed and distributed within the EU by Dentsply Sirona but showing the manufacturer to be Maruchi. Another example from this field is TotalFill BC by FKG Dentaire, which is manufactured by Innovative Bioceramix in Canada and sold under the EndoSequence BC brand in the United States (21).

The authors assume that in the future dental companies selling private label products will not act as manufacturers, but will rather declare the OEM as the manufacturer according to MDR on the label and, thus, might act as a distributor instead of being the manufacturer. Along with this, the OEM does not have to provide access to the technical file of the product.

These changes could have a positive effect for dentists, who can now identify products of identical composition, which were marketed under different brand names, and could make a purchase decision purely based on economic reasons or utility, rather than marketing. Also, the discontinuation of the private label manufacturing setup could shine a new light on the vast amount of material comparison studies in dentistry. Researchers might identify products that they tested from different brands, which were actually produced by the same OEM, and thus were one and the same.

## Research implications

Dental researchers, especially those in materials science, have traditionally been looking to create intellectual property for their respective schools (22). In dentistry as well as in other fields with a focus on medical devices, the metaphorical gap between the laboratory and the clinic has perhaps seemed less wide than in other medical subjects, in which pharmaceuticals are the key elements. Therefore, working in the dental materials field, apart from creating new knowledge, had two additional elements for researchers, namely the possibility to gain third-party money from the dental industry to develop new devices, and secondly, the opportunity to get a job in that industry in a Research and Development unit. However, the environment that created access to industry funds and employment may change soon or already has changed in Europe because of the MDR. The manufacturers of medical devices will have to evaluate their newly developed technologies more rigorously, their return on investment will be lengthened, and there will be less money in the pot to support university research. Moreover, within the companies themselves, Research and Development units will lose importance compared to the Regulatory teams.

As mentioned above, the clinical evaluation of devices will play a larger role in the conformity assessment procedure. Manufacturers might be forced to restrict the claims on intended use, due to a lack of sufficient clinical data. For example, Mineral Trioxide Aggregate (MTA) was and is advocated for various indications such as apexification, root end filling, perforation repair and others (23). Under the old legislation (MDD), MTA manufacturers often referred to competitors' products and claimed equivalence due to the similar nature and composition of MTA materials. However, this will not be possible anymore under the MDR and each manufacturer has to show sufficient clinical data for their product and its respective indication. The equivalence claim can only be applied under specific conditions (18). If this data is not available, a manufacturer might have to restrict the intended use of a product. On the other hand, the new regulatory environment could also lead to engaged manufacturers who are willing to perform more clinical investigations with dental researchers to extend the claims of a device and have an advantage over competitors.

Another opportunity for dental researcher arises from the need of notified bodies to have access to qualified experts to perform the conformity assessment (Annex VII, MDR). A dental researcher and medical doctor can be valuable to the notified body as he/she can evaluate not just the technical file but also the clinical evaluation report or the post-market clinical follow-up plan and report, where discipline-specific knowledge is needed. This opens new employment opportunities for dental researchers next to a purely academic career.

## Dental clinics

The main MDR-related impact on all dentists and their clinics concerns infection control, or more precisely, the sterilization of dental instruments intended for repeated use in patients (24). This is specified in EN ISO 17664. Dental mirrors, probes, burs, etc. all are medical devices, as are dental autoclaves and other sterilization equipment, which also fall under the MDR. Under this new regulation, there needs to be improved designation of dental instruments as being sterile. In theory, each individual instrument used in a patient has to be traceable to the respective sterilization process [Annex I 23.4 (n), Annex VI, part C (4.10), MDR]. In the case of dental burs, this means that they either have to be imprinted with an individual UDI or be designated as single-use. Both concepts, if enforced completely, would drive up costs for dentists and patients considerably. Furthermore, there is a requirement for validation and routine control of the sterilization process itself, and the implementation of sterile barrier systems (provision of ISO 13485:2016) that maintain the sterility of a device to the point of use (24). The MDR has changed “devices delivered in a sterile state” to “devices labelled as sterile”. This means that the end user has to control packaging.

The main burden will again be on the manufacturers of sterilization units, who have to provide their customers, i.e., the dentists, with the respective software and hardware to be able to comply with the MDR. This also means that the MDR will most likely create good business for the manufacturers of dental autoclaves in the mid-term, because the old sterilizing equipment lacks the now necessary wholistic documentation features. Dentists will be confronted with yet more administrative work, and procedure prices will invariably go up. However, how, when, and in which EU countries these new MDR requirements and the issues discussed below will be imposed and monitored in dental practice is an open question.

A second important MDR-related novelty for dentists relates to computer-aided design and manufacturing (CAD/CAM). The dentist who owns and uses a CAD/CAM outfit fulfills the definition of being a manufacturer according to the MDR [Art. 2 (30)], because the respective restorations are indeed created on site. These manufactured devices are so-called “custom-made” [Art. 2 (3), MDR] as confirmed by the European Federation of Laboratory Owners and Independent Dental Technicians (FEPPD) who requested a legal opinion from the EU Commission. Hence, dentists who will carry out the CAD/CAM manufacturing themselves are obliged to comply with the MDR as manufacturers, similar to dental laboratories. For both these parties, there are less stringent obligations laid out as compared to industrial manufacturers as they merely manufacture custom-made devices. Nevertheless, certain requirements need to be fulfilled also by the manufacturer of custom-made devices (Art. 10, MDR). A quality management system needs to be in place, which defines responsibilities and processes [Art. 10 (9), MDR]. Additionally, a risk management system needs to be implemented. The MDR has a dedicated section for custom-made devices, Annex XIII. Among other items, it states that manufacturers shall draw up a declaration of conformity, keep this declaration of conformity for a period of at least 10 or 15 (implantable devices) years and perform a surveillance of the post-production phase of the device. When manufacturing CAD/CAM in the dental practice, dentists shall be aware that they have to fulfill the obligations as set forth within the MDR.

Another issue that has not been openly discussed in this context is the clinical application by dentists of materials available from sources other than medical device manufacturers. These include, but are not limited to, sterilized Portland cement from the hardware store as an alternative to MTA (25), sodium hypochlorite in the form of household bleach (26), or calcium hydroxide powder from a chemical supplier. It should be clear that, when applying such materials to patients, the dentist bears the full legal responsibility and liability for any untoward effects and, depending on national law, commits a criminal act (27). Therefore, and despite a clear legal framework covering this issue in Europe at the time of this publication, such practice should be advised against in the ever-evolving environment of maximum patient protection and full treatment documentation (28). To a lesser extent, these concerns can also be extended to medical devices ordered from the local pharmacy upon a prescription written by the dentist. In that case, the source of the device could be more reliable, yet the dentist has to bear the brunt of legal responsibility.

Last but not least, and as described above, old devices with a long history of clinical use but without sufficient clinical data could potentially disappear. As the CED stated in a recent press release, up to 35% of devices could perish because the devices will not be conform to MDR, even though they have a proven history of use without any risks or incidents, yet lack the required clinical data. If the requested clinical studies are not economically feasible the manufacturers might not re-certify their device, which will then ultimately not be available to European dentists and their patients. The CED called in their statement for a permanent extension of MDD certificates for devices that have been on the market for years and are considered safe and reliable (11). Whether this call is heard by the EU Commission or Parliament remains elusive at the time of writing this article, but the before-mentioned proposed extension could provide some time to resolve the matter.

## Conclusions and outlook

Although the MDR covers more than 200 pages, including 17 annexes, further guideline documents are needed, published by the MDCG of the EU Commission several times a year. These MDCG-endorsed documents aim to show the practical application of the MDR, yet they are not legally binding. Nevertheless, manufacturers as well as notified bodies rely and refer to them.

In summary and based on current knowledge and experience, it may be stated that, based on the increased regulatory red tape, the MDR creates higher costs to the dental industry for each of their products that they want to maintain in the European market. Logic would dictate that, therefore, less money can be spent on research by these commercial enterprises, and their focus has shifted from novelty to safety and market access. On the other hand, and as delineated above, the MDR may also create new career opportunities for dental researchers. To European dentists, the new regulation also inflicts a higher workload and need to self-monitor and document.

Only the future can show whether patient protection will really be improved by the complex set of regulations that is the MDR, or whether the MDR will even harm patients by the invariably reduced number of medical devices that will be available. In the US, patients can be protected directly by the Park doctrine (29). Medical device executives who made revenues on devices that violated federal law can be held accountable directly. However, a reform of medical device guidelines is also called for in the United States, where, for example, avoidable deaths occurred with reperfusion catheters (30). Therefore, the MDR may act as an antetype for similar regulations in rule-of-law countries and their respective markets around the world. There will be less products, but these will probably be safer and better documented.
